# Ultrasensitive Detection of C-Reactive Protein by a Novel Nanoplasmonic Immunoturbidimetry Assay

**DOI:** 10.3390/bios12110958

**Published:** 2022-11-02

**Authors:** Tang Dang, Zhenyu Li, Liyuan Zhao, Wei Zhang, Liping Huang, Fanling Meng, Gang Logan Liu, Wenjun Hu

**Affiliations:** 1 School of Life Science and Technology, Huazhong University of Science and Technology, Wuhan 430022, China; 2 Department of Bioengineering, The University of Tokyo, 1-3-7 Hongo, Bunkyo-ku, Tokyo 113-8656, Japan; 3 Cancer Center, Union Hospital, Tongji Medical College, Huazhong University of Science and Technology, 1277 JieFang Avenue, Wuhan 430022, China; 4 National Engineering Research Center for Nanomedicine, College of Life Science and Technology, Huazhong University of Science and Technology, Wuhan 430074, China

**Keywords:** ultrasensitive detection, surface plasmon resonance, nanophotonics, C-reactive protein, one-step detection, rapid diagnostics

## Abstract

Nanotechnology has attracted much attention, and may become the key to a whole new world in the fields of food, agriculture, building materials, machinery, medicine, and electrical engineering, because of its unique physical and chemical properties, including high surface area and outstanding electrical and optical properties. The bottom-up approach in nanofabrication involves the growth of particles, and we were inspired to propose a novel nanoplasmonic method to detect the formation of nanoparticles in real time. This innovative idea may contribute to the promotion of nanotechnology development. An increase in nanometer particle size leads to optical extinction or density (OD)-value changes in our nanosensor chip at a specific wavelength measured in a generic microplate reader. Moreover, in applying this method, an ultrasensitive nanoplasmonic immunoturbidimetry assay (NanoPITA) was carried out for the high-throughput quantification of hypersensitive C-reactive protein (CRP), a well-known biomarker of cardiovascular, inflammatory, and tumor diseases. The one-step detection of the CRP concentration was completed in 10 min with high fidelity, using the endpoint analysis method. The new NanoPITA method not only produced a linear range from 1 ng/mL to 500 ng/mL CRP with the detection limit reduced to 0.54 ng/mL, which was an improvement of over 1000 times, with respect to regular immunoturbidity measurement, but was also effective in blood detection. This attractive method, combined with surface plasmon resonance and immunoturbidimetry, may become a new technology platform in the application of biological detection.

## 1. Introduction

Recently, nanotechnology has been focused on and studied extensively. The fascinating and unparalleled properties of nanostructured materials and devices continue to open up new and sometimes unexpected applications. It has been widely applied in various fields including healthcare, medicine, food, nutrition, transportation, energy, environment and many more application scenes [1,2,3,4]. Nanofabrication methods can be categorized into the top-down and bottom-up approaches [5]. The top-down method uses a photolithography process to etch bulk materials, to achieve the required smaller nanostructures [6]. By contrast, the bottom-up approach involves crafting the structures, molecule by molecule, through covalent or electrostatic interactions [6]. Moreover, if the self-assembly process from small to large molecules can be monitored in real time, the conditions, rate, and status of the bottom-up process can be analyzed, thus making it possible to control the proportion of reagents added and the conditions of the reaction better. Enhanced nanomanufacturing processes may lead to broader and deeper applications of nanotechnology. As a result, it is important and essential to find an innovative method of monitoring the nanomanufacturing process in real time.

In the last decade, biosensors based on localized surface plasmon resonance (LSPR), which is an optical phenomenon caused by the collective oscillation of electron gas in a metal nanostructure surrounded by dielectric material, have been studied extensively, because of their unique benefits such as being low cost, having higher surface-to-volume ratio, and not requiring any prisms [7,8].They mainly consist of nanoparticles of gold [9], silver [10] and metallic periodic nanostructures such as nanoholes and nanoslits [7,11]. The principle of LSPR is sensing the refractive index change near nanoparticles or nanostructures, due to the binding of biomolecules. Moreover, it is a method that can quantify biomolecules by the amount of change in the intensity of resonance peaks of reflection or transmission spectra or the shift of resonance wavelengths. In particular, when nanoholes are periodically manufactured over a large area, the surface plasmon polaritons can be excited, so that extraordinary optical transmission (EoT) is generated through the nanoholes at a specific resonance wavelength [12,13]. Therefore, it is possible to use the generic microplate reader as a detection medium.

The C-reactive protein (CRP), the first acute-phase protein produced by the liver, is a sensitive systemic marker of inflammation and tissue damage [14]. Serum CRP levels could increase as much as 10,000 fold in acute response to serious infection or major tissue damage [14,15]. It is a well-known biomarker, widely used in detecting infections and inflammatory conditions such as cardiovascular diseases [16], myocardial infraction [17], stroke [18], pneumonia [19], and cancer [20,21]. In addition, the hypersensitive C-reactive protein (hsCRP) is one of the more specific markers of atherosclerotic vascular disease. Early detection and prevention are extremely important for improving survival rate of patients [22]. The normal blood level of CRP has been reported to be less than 2.5 μg/mL, and meanwhile, concentrations of CRP higher than 5 μg/mL indicate a pathological condition [23]. Therefore, developing a rapid, selective, and precise method for hsCRP detection plays a pivotal role in early screening for diseases.

**Figure 1:**
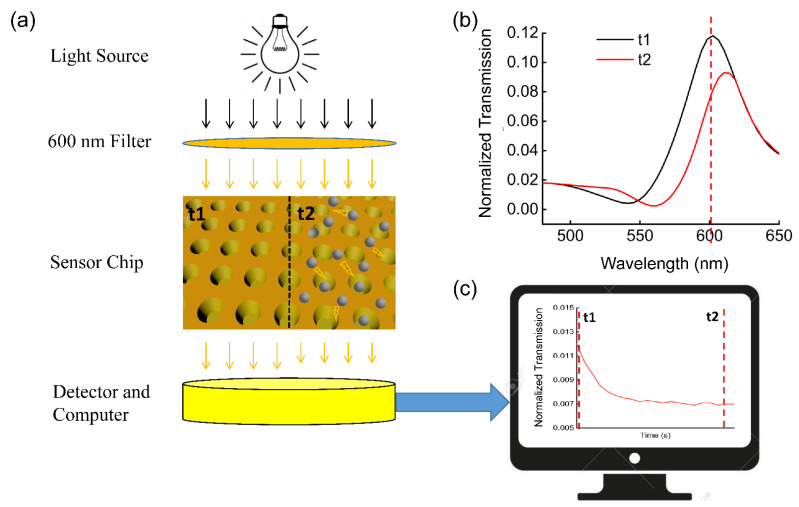
(**a**) The process of precipitation complex detection (t1: the spectrum before the complexes appear; t2: the spectrum after the complexes appear). (**b**) Spectra graph before and after the appearance of the compound detected by the spectrometer. (**c**) Detecting the process of complex formation by using 600 nm filter.

In this study, we demonstrated a novel nanoplasmonic method based on the EoT, which can detect the formation of nanoparticles in real time, and the general principle is shown in Figure 1a. This configuration mainly consists of a light source, filter, and detector, etc., and can be directly integrated into the microplate reader (XLEMENT WeSPR 100 Multifuncitonal Molecular Analyzer, Shanghai, China). Therefore, it eliminates the need for expensive and large spectrometers, which allows it to be more compact and economical. Figure 1b shows the spectra read by a microplate reader before and after the compound was added into the background buffer. We can see the light intensity shows a significant drop at the wavelength of 600 nm. We applied this method to three experiments, with increasing experimental complexity: detecting calcium carbonate (CaCO_3_) trace precipitation, self-aggregation of human islet amyloid polypeptide (IAPP), and ultrasensitive C-reactive protein (CRP). As the size and number of nanoparticles increase, the transmission spectrum of the LSPR chip changes, which enables the change process of the nanoparticles in the solution to be recorded as shown in Figure 1c. In addition, the changes in transmission intensity can be measured through the optical density (OD) variation of the nanosensor chip with a microplate reader, and the relationship between them can be expressed as $\mathrm{OD}\;\mathrm{value}=-\log_{10}\left(\mathrm{Transmittance}\right)$ [24]; therefore, in the Experimental Section, we will record the OD value instead of the transmittance, at 600 nm wavelength. Firstly, we used the well-known compound CaCO_3_ to verify the feasibility of this method, preliminarily. We then detected the self-aggregating molecules and the content of the biomolecules. Therefore, for the detection of CRP, the ultrasensitive nanoplasmonic immunoturbidimetry assay (NanoPITA) method which combines the LSPR and immunoturbidimetry has been demonstrated. The results showed that NanoPITA can distinguish 1 ng/mL of CRP in only 10 min, without complicated operations, and is 1000 times more sensitive than the conventional immunoturbidimetric method. A detailed explanation of the relationship between the NanoPITA method and immunoturbidimetry is shown in Figure S1 and Supplementary Information. In short, the detection is expected to proceed in the antibody excess zone, where the antibody–antigen precipitation has positive correlation with the antigen. At the present time, the conjugations of the reagent with nanomaterials such as latex particles [25,26] and nanogold [27,28] have been made, to enhance immunoturbidimetry sensitivity, but the biochemical conjugation process is relatively complicated. The convenient, simple and easy-to-operate NanoPITA method can be more suitable for ultrasensitive, rapid, and accurate analysis of protein markers, showing great potential in early clinical diagnosis of cardiovascular disease.

## 2. Experimental Methods

### 2.1. Immunoturbidimetry Sensor Fabrication

The fabrication of the sensor has been described in detail in our previous research [29]. In brief, the sensor was fabricated by a nano-replication process. The original silicon mold with a tapered nanopillar array was put into a vacuum dryer full of Hexylsilane (Sigma Aldrich, Shanghai, China) for 12 h, to enhance hydrophobicity, and then Norland optical adhesive (NOA-61, Sigma Aldrich, Shanghai, China) was spread evenly on the mold. Next, a polyethylene terephthalate (PET, Sigma Aldrich, Shanghai, China) sheet was placed on top of it. After 3 min of UV light irradiation, the PET sheet with the optical adhesive layer was peeled off. Then, 9 nm-thick titanium (Ti) and 110 nm-thick gold (Au) were sequentially deposited onto the nanocup array surface of the optical adhesive layer. The sheet was later cut into small pieces of 1 cm × 1 cm sensor chips, which were glued onto the bottom of a through-hole 96-well plate made by a 3D printer (Object 30 primer^TM^, Stratasys Ltd., Eden Prairie, MN, United States.) and called SPR chips.

### 2.2. Detection of Calcium Carbonate (CaCO_3_) Trace Precipitation

The formation of calcium carbonate is a very famous experiment in chemistry textbooks, due to its low solubility in water [30]. However, when the concentrations of calcium chloride (CaCl_2_) and sodium carbonate (Na_2_CO_3_) are very low, the occurrence of precipitation cannot be observed with the naked eye or even under a microscope. Here, we used 10 mM Na_2_CO_3_ and 0–10 mM CaCl_2_ (Sigma Aldrich, Shanghai, China; diluted with deionized distilled water (DDW)), to perform the experiments. We added 30μL Na_2_CO_3_ and CaCl_2_ solution directly onto SPR chips, and quickly put them into the microplate reader (XLEMENT WeSPR 100 Multifuncitonal Molecular Analyzer, Shanghai, China). We then measured the dynamic absorption spectrum, over time. The microplate reader was set to read every 30 s for 10 min, with no oscillation mode.

### 2.3. Detection of Self-Aggregation of Human IAPP

IAPP is named for its tendency to aggregate into insoluble amyloid fibrils, which have cytotoxic properties and are believed to be of critical importance in type 2 diabetes [31]. In addition, the IAPP can precipitate spontaneously as amyloid, in vitro [32]. Amyloid is a general term for a special state of protein aggregation, and in this state, β-sheet-shaped molecules bind to each other mainly through hydrogen bonds [33,34].

Usually, the IPPA powder is stored at −20 °C. Before the experiment, we added 800 μL Tris (Tris(hydroxymethyl)aminomethane hydrochloride) buffer (Sigma Aldrich, Shanghai, China), to dissolve the IPPA powder; the concentration of IPPA in this solution was 32 μM. In addition, other concentrations of the IPPA were quickly prepared and added to a 96-well plate for kinetic detection. The microplate reader was set to read every 1 min for 5 h, with no oscillation mode.

### 2.4. NanoPITA of CRP

The NanoPITA CRP detection was carried out with a microplate reader. First, we added 100 μL 30μg/mL bovine serum albumin (BSA) solution (Sigma Aldrich, Shanghai, China) (20~22 °C) for 30 min, to reduce the adsorption of protein molecules on the surface of the SPR chips. Next, we mixed a different concentration of 10 μL CRP with 20 μL 10% PEG-6000, and kept the mixture at room temperature for 5 min. The PEG-6000((Sigma Aldrich, Shanghai, China)) was added to accelerate the immunoprecipitation formation, which made the detection faster. We then threw away the BSA solution, and washed the sensor chip 2 times with buffer, put 30 μL 30 μg/mL anti-CRP antibody onto the sensor chip, and let it sit for 5 min. Finally, the CRP samples with PEG-6000 were mixed on the chip and the dynamic absorption spectrum was measured over time. In addition, to reduce the time difference caused by the sample addition, we used a multi-channel pipette to add it to the SRP chip, to avoid the influence caused by sample-addition time. The dynamic measurement frequency and period were set to 30 s for a total of 10 min, with normal oscillation mode (Frequency: 9.2 Hz). Setting the normal oscillation mode can improve the diffusion between biomolecules, which can make CRP and CRP antibodies bind faster to form complexes, thereby reducing the time for the reaction to reach saturation. The buffer solution for all reagents was 20 mM Tris-HCl. The wavelength used for the analysis was 600 nm.

### 2.5. FDTD Simulations

We performed three-dimensional finite-difference time-domain (3D-FDTD) simulations using commercial software (FDTD solutions, Lumerical, Vancouver, Canada). The model had the same geometry as the actual sensor device. In particular, the top and bottom diameters of the nanocup were 200 nm and 180 nm, respectively, the depth of the nanocup was 500 nm, and the periodicity of the cup was 320 nm. The refractive index of protein is related to the type and concentration of the protein, and it ranges from 1.515–1.648 [35]. In this experiment, we used spheres with a refractive index of 1.6 to approximately simulate the immune molecular complex. The background refractive index was set to 1.333 to simulate a liquid environment. The models had the same nanocup structure but the diameters of the immune molecular complex were different. The sensor chips were illuminated with a plane wave from the top side (z direction). A perfect matching layer was applied to the boundary conditions in the z axis, and a periodic one was applied to the boundary conditions in the x and y axes.

### 2.6. Immune Molecular Complex Size Determination

The size of the immune molecular complex was determined by dynamic light scattering analysis (Zetasizer Nano ZSP, Malvern, England); 3 × 3 measurements were performed at 25 °C. For every measurement, the auto correlation function was able to ensure the sample quality. Additionally, the size was determined by transmission electron microscopy (TEM, HITACHI 7700, Hitachi, Tokyo, Japan). The sample was dropped onto a copper grid, and after 2 min, the excess solution was removed with filter paper. Then, 1% uranium acetate was dropped onto the copper grid, and the remaining procedures were the same as described earlier. The image of the dried sample was taken with a TEM (HITACHI 7700, Hitachi, Tokyo, Japan).

## 3. Results and Discussions

### 3.1. Device Characterization

**Figure 2:**
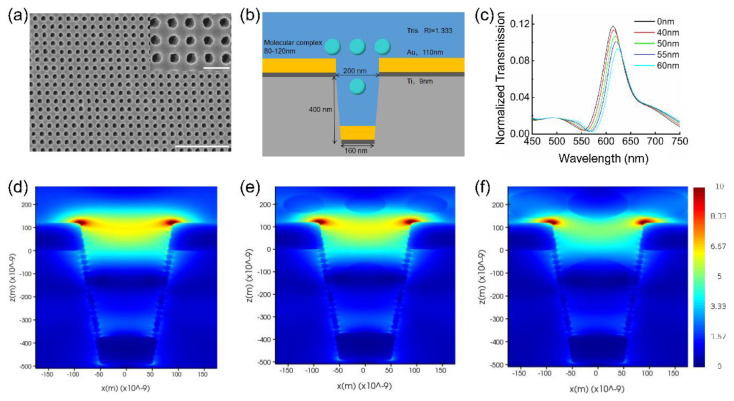
(**a**) SEM image of the fabricated nanocup sensor structure (Scale bar: 3000 nm). Inset: at high magnification (Scale bar: 500 nm) (**b**) Simulation model of the nanocup plasmonic sensor. (**c**) Simulated transmission spectra of the nanocup with different sizes (radius = 0, 40, 50, 55 and 60 nm) of immune complex molecules. (**d**–**f**) Near-field electric field distributions of plasmonic nanocup at resonance wavelength with immune complex particles at a radius of 0 nm (**d**), 40 nm (**e**), and 60 nm (**f**).

To investigate the mechanism of immunoturbidimetry signal sensing in the nanocup sensor, we used the 3D-FDTD method to simulate the optical transmission spectra with no immune complex molecule, and varied the size of the immune complex molecules. However, in order to perform the FDTD simulation with more accurate parameters, we first characterized the chip and the antigen–antibody complex. The scanning electron microscopy (SEM) image of the fabricated nanocup sensor-structure is shown in Figure 2a. We can see that the sensor chip has a high uniformity cup-array structure with similar morphology, and through the high magnification of SEM images, we know the top diameter of the nanocup is ~200 nm, with a periodicity of ~360 nm. In addition, the sizes of the immune complex particle with different concentrations of the CRP are measured by the TEM and dynamic light scattering analysis. Figure S2a (Supplementary Information) shows the TEM image after 15 min of 500 ng/mL CRP, anti-CRP antibody, and PEG-6000 reaction. Although the particle size shown by TEM is not uniform, we can see that the diameter of the immune complex molecule is ~100 nm to 120 nm. The reason for the inhomogeneity may be due to the reaction in the 1.5 mL centrifuge tube, but it is more conducive to the formation of uniformly sized particles if reacting in a 96-well plate with the help of shaking. Similar results can be observed with higher concentrations of the CRP in Figure S2b. The final average diameter of the immune complex molecule is ~100 nm, only when the CRP concentration is 5 ng/mL or lower (average particle size is ~80 nm), since the reaction has not reached its full equilibrium state.

The simulation model of the nanocup based on SEM and TEM images is shown in Figure 2b. As the optical electric fields are localized to within ~250 nm from the gold surface in SPR configuration [36], we only consider the complexes in the effective area, including 250 nm above the gold film, and in the nanohole area, as the area in the nanohole will affect the change of the transmitted light. As the composites are uniformly distributed, their ratio of the composites in the effective area to those in the nanohole area, should be the same as their volume ratio. We roughly calculated the volume of the two areas in one period. The calculated volume of the effective area and the nanohole area are 9.1 × 10^6^ nm^3^ and 2.54 × 10^7^ nm^3^, respectively, and the ratio of effective area to nanohole area is approximately 2.7. In the condition of homogeneous solvent, the simulation results show that three nanoparticles were located outside the nanocup while one nanoparticle was located in the nanocup. The diameters of the nanoparticles were changed from 80 nm to 120 nm, and the result is shown in Figure 2c. It indicates that transmitted light intensity decreases at the 610 nm (peak without nanoparticle), and there is an overall redshift of the peak as the nanoparticle size increases, which is consistent with other studies on SPR sensing of precipitation [37]. To clarify this phenomenon, the near-field electric field distributions of the immune complexes with different radii were simulated, and the results are shown in the Figure 2d–f. According to the near-field electric field distribution figure, as the size of the immune complexes increase, a part of the electric field will be transferred from the chip (Au) to the immune complexes, resulting in a decrease in the electric field strength at the edge of the nanocup; this is also the fundamental reason for the weakening of the transmitted light. In addition, because the refractive index of the immune complex (1.6) is larger than that of water (1.333), when the immune complex is close to the nanocup, the LSPR phenomenon appears, which is similar to biomolecules attached to gold nanoparticles [38,39], that is, the redshift of the resonance peak also occurs. However, the spectrum without any immune complex nanoparticle measured by a spectrometer is shown in Figure 1b, and it shows that the resonant wavelength of the sensor chip is near the 600 nm point. Spectral differences between the experimental results and the simulation may be due to variations in the manufacturing process. In subsequent experiments, we measured and analyzed the optical density (OD)-value change at 600 nm over time.

### 3.2. Detection of CaCO_3_ and Self-Aggregation of The IAPP

**Figure 3:**
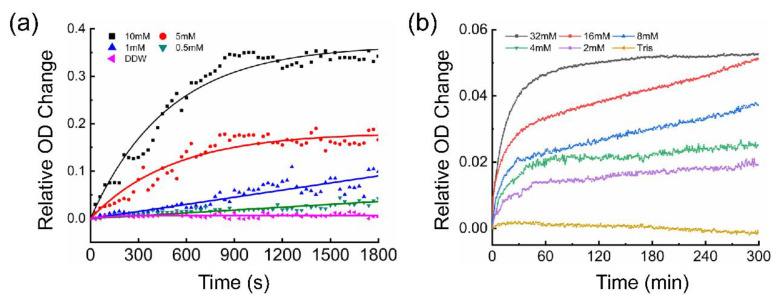
(**a**) Dynamic curve of the reaction of different concentrations of CaCl_2_ and 10 mM Na_2_CO_3._ (The solid lines represent the fitting curves.) (**b**) Dynamic curve of self-aggregation of different concentrations of the IPPA.

Here, the most common CaCO_3_ experiment is used to verify that NanoPITA can detect the formation process of tiny amounts of precipitation. The result is shown in Figure 3a. We can see the OD value increases with time at the 600 nm wavelength, due to the formation of CaCO_3_. Moreover, the OD-value change is greater when there is a faster formation rate of CaCO_3,_ caused by higher concentration of CaCl_2_. In the low concentration range (less than 1 mM), the precipitation cannot be directly observed with a microscope (the microscope images of different concentrations after 30 min of reaction are shown in Figure S3a–e). Since the resolution of a general optical microscope is ~200 nm [40], the particle size of the CaCO_3_ compound formed at low concentrations may be lower than 200 nm.

Figure S3f shows the average gray value of these microscopic images. The gray value of 10 mM CaCl_2_ and 5 mM CaCl_2_ are obviously smaller than that of the DDW, but the difference among 0.5 mM CaCl_2_, 1 mM CaCl_2_ and DDW is not obvious. This result implies that the proposed method can detect the low content of heavy metals in the environment.

Figure 3b shows the OD change in the self-aggregation of the IAPP, which is a substance that can quickly accumulate in vitro. The OD value does not change much in the absence of the IAPP, while the OD value of high-concentration IAPP increases rapidly, due to the occurrence of aggregation. In addition, we detected the particle size of 32 mM IPPA self-aggregation reaction for 1 h and 5 h by dynamic light scattering analysis, and the results are shown in Figure S4. It shows that particle size of the reaction for 1 h and 5 h is very different, but the OD change in the transmitted light is insignificant. This phenomenon may imply that when the diameter of the composite is around 100 nm, the reaction may reach its limit (saturation), and continuing growth of the composite will not cause too much light-intensity change.

### 3.3. NanoPITA of CRP

Before the detection, the sensor chips were washed with 70% ethanol and deionized water, to remove any impurities on their surfaces. Different concentrations of the CRP and PEG were mixed in Eppendorf (EP, Eppendorf, Hamburg, Germany) tubes and kept at room temperature for 5 min. We then added the mixtures and the CRP antibody on the sensor chips, while the measurement of the reaction kinetics started at the same time. More detailed information is provided in the Methods Section. The PEG helps to decrease the solubility of the protein molecules, drive the antigen–antibody interaction toward immunoprecipitation formation [41], and accelerates the immunoprecipitation formation, thus making the detection time shorter. As the reaction progressed, 90–120 nm immune molecular complex particles gradually formed, and resulted in the OD value changing at 600 nm.

**Figure 4:**
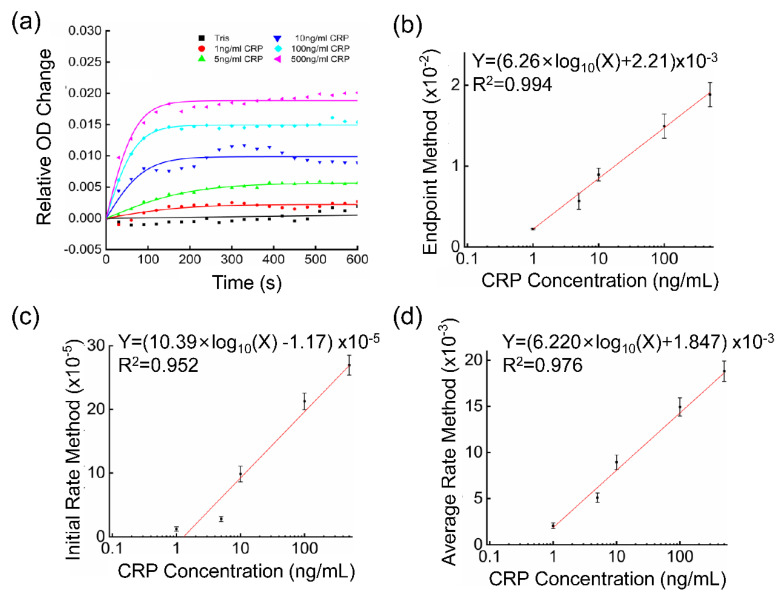
(**a**) Plot of the relative OD value change at the 600 nm wavelength for different concentrations of CRP sample. (**b**–**d**) Line fitting showing the relative OD value change for different concentrations of CRP sample by using (**b**) endpoint analysis method (value of $\frac{A}{2}$), (**c**) initial rate analysis method (value of $\frac{A\times B}{4}$), and (**d**) average rate analysis method.

As shown in Figure 4a, we can distinguish between samples with different concentrations of the CRP from 1 ng/mL and 500 ng/mL. The concentration of the CRP in the blood of a healthy person varies from 0 to 5 μg/mL [42]. It has been reported that the concentration of the CRP in human saliva is 200 times lower than that in the serum [43]. Therefore, the sensor chip may be applied to detect the CRP in saliva. To reduce errors and provide quantitative standards, the plots in Figure 4a were fitted with a sigmoid-like function $y=A\left(\frac{1}{1+e^{-Bx}}-\frac{1}{2}\right)$. The specific information about the fitting can be found in Table S1. At the beginning of the kinetic reaction (x = 0), the relative OD change was 0. Therefore, the constant −1/2 was used to adjust the initial value. The parameter “A” reflects the relative OD change in the final steady state, and the parameter “B” partly reflects the rate at which the reaction reaches its equilibrium state.

### 3.4. Quantitative Analysis of the Kinetic Results

The concentration of CRP in the ordinary immunoturbidimetry method can be determined by the initial rate analysis, average rate analysis, or endpoint analysis [44]. Likewise, in the NanoPITA method, the concentration of CRP can be measured by the endpoint analysis, initial rate analysis, and average rate analysis.

For the endpoint analysis, the relative OD changes were compared when all the reactions with different concentrations of the CRP reached the equilibrium. Under the equilibrium circumstances, $y=\frac{A}{2}$ and the result was only relevant to parameter “A”. The reaction endpoint result or the parameter “A” are shown as the function of different concentrations of the CRP in Figure 4b. The results were fitted to an exponential function, and they were consistent from 1 ng/mL to 500 ng/mL. The coefficient of determination (R^2^) for the fitting was calculated to be 0.994, which meant that the correlation was nearly perfect, and the concentration of the CRP can be quantified better with this method. Under these circumstances, the calibration equation can be expressed as the concentration of CRP $10^{\frac{100\times A-0.442}{1.252}}$.

Therefore, if we choose maximum reaction rate to quantitate our samples, the initial rate should be identified as the maximum reaction rate, and analyzed by differentiating the equation when time(x) equals 0, $y=\frac{A\times B}{4}$. Figure 4c shows the relationship between the initial rate and different concentrations of the CRP, with fitting result R^2^ 0.952. The reason for the lower fitting may be due to the time delay between the measurement and reaction starting-time points. Finally, we calculated the average rate in 5 min with the fitting result R^2^ 0.976, which is shown in Figure 4d. The formula LOD = 3.3 σ/S is utilized to calculate the LOD for different methods, where S is the slope of the calibration curve and σ is the standard deviation of the response. Through calculation, the LOD of the endpoint analysis method, the maximum reaction rate method and the average rate method were obtained as 0.54 ng/mL, 1.35 ng/mL and 0.75 ng/mL, respectively.

It is most effective to use the endpoint method to quantify the CRP for the NanoPITA method, due to its better fit coefficient and lower LOD. It could be explained that the final immunoprecipitation content is more impactful than the rate of immunoprecipitation formation. Therefore, the endpoint method will also be used to quantify CRP for blood samples, in Section 3.5.

The LOD, process time, and biological modification time in our present methodology were compared with other methods reported in the literature in Table 1. It can be seen that for all the processes, NanoPITA, one-step assay without tedious operations (only 35 min biological modification + 10 min detection time), is extremely sensitive (LOD below 1 ng/mL) for CRP detection within 1 h. Compared to biosensors with a long biological modification time, it is generally a method costing only little time for pre-modification, cryopreservation, and renaturation when it is used. However, the inevitable problem is that the longer it is stored, the more degradation of antibodies, proteins or nucleic acids on the chip surface, appears. Conversely, detection could start from the biomodification steps and finish completely in several hours. Therefore, this new method with the SPR nanochip provides great potential for the ultrasensitive detection of specific protein biomarkers.

### 3.5. Evaluation of Enhancement and Cross-Reactivity

**Figure 5:**
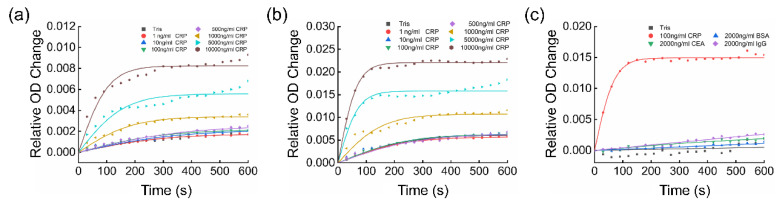
Plots of the relative OD change at (**a**) 600 nm and (**b**) 340 nm in regular immunoturbidimetry assay of CRP samples in a regular microplate without integrated nanosensor chips. (**c**) Plot of the relative OD change at the resonant wavelength 600 nm using NanoPITA for the CRP, BSA, CEA and IgG in a microplate with integrated nanosensor chips.

To evaluate the enhancement by the nanoplasmonic device, we carried out comparative immunoturbidimetry experiments in an ordinary 96-well plate without the SPR sensor chip, using the same microplate reader. The reaction kinetics measurement results at the resonant wavelength (600 nm) of the SPR sensor chip and at the wavelength (340 nm) for regular immunoturbidimetry [50] are shown in Figure 5a,b, respectively. The specific information about the fitting is presented in Tables S2 and S3. From both of the results, we know that the immune complex molecules have greater absorption at 340 nm, and the regular immunoturbidimetry at either 600 nm or 340 nm can only distinguish CRP concentrations higher than 1 μg/mL. In comparison, our NanoPITA measurement results indicate that the LOD is below 1 ng/mL with the SPR sensor enhancement, which is an improvement of over 1000 times, with respect to regular immunoturbidity measurement under the same conditions.

The cross-reactivity or specificity of NanoPITA was evaluated by comparing the relative OD changes with the samples of 100 ng/mL CRP, 2000 ng/mL bovine serum albumin (BSA), 2000 ng/mL carcino-embryonic antigen (CEA), and 2000 ng/mL immunoglobulin G (IgG). As shown in Figure 5c, the NanoPITA method reveals obvious signal responses to 100 ng/mL CRP, while much lower responses to other nonspecific proteins, similar to the level of the Tris buffer. Table S4 shows the specific information about the fitting. The comparative experiment proves that the immunoturbidimetry with SPR nanosensor enhancement or NanoPITA has good specificity and sensitivity.

### 3.6. Evaluation of Blood Sample

**Figure 6:**
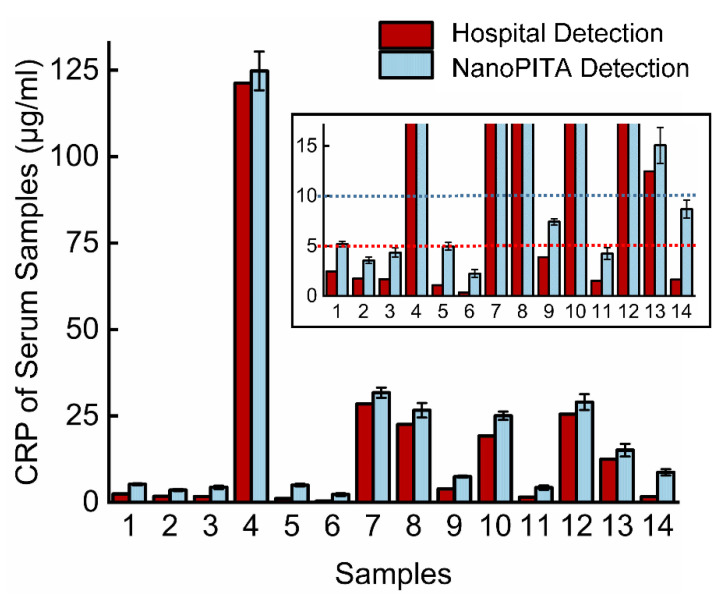
Measurement of CPR with different blood serum samples by the NanoPITA method in the lab and latex agglutination in the hospital. The figure on the upper right is a close-up view of the low concentration, where the red and blue dotted lines represent 5 μg/mL and 5 μg/mL, respectively.

The blood sample acquisition and processing methods have been described in our previous research work [29]. Specifically, the blood samples were centrifuged at 2500× *g* and 4 °C for 15 min, and then the sediment was discarded. We took the supernatant solution and stored it at −80 °C, for subsequent use. For the experiment, first, the serum samples were put in a water bath at 37 °C for 10 min, and then the samples were diluted 100 times with 20 mM Tris-HCl. (Among them, sample No. 4 was diluted 500 times, because the first measurement result after 100 times-dilution was above the 500 ng/mL). Fourteen blood samples were quantified in our lab and in the Wuhan Union hospital. All subjects gave their informed consent for inclusion before they participated in the study. The study was conducted in accordance with the Declaration of Helsinki, and the protocol was approved by the Ethics Committee of IEC(A239). The results of the 14 samples are shown in Figure 6. We can see that the results from the NanoPITA method are slightly higher than those from the hospital. This is because the blood samples are not the same as pure samples; even if the serums are diluted 100 times and the chip has been blocked with BSA, the protein in the serums would inevitably be non-specifically adsorbed onto the chip surface, which would also lead to an increase in the OD value. Therefore, we might need a new and powerful blocking reagent or some data-processing arithmetic to eliminate the non-specific signal. The result shows that the NanoPITA method is practical for CRP detection in clinical rapid diagnostics, even if it gives a slightly higher value. Subsequent experiments will focus on how to employ suitable surface modifications to inhibit nonspecific binding in blood.

## 4. Conclusions

This work proposed a novel method to detect the formation of nanoparticles in real time by SPR nanosensor-integrated microplate wells using a generic microplate reader. Moreover, we successfully applied this method to biosensors to detect the biomarker CRP. Characterized by DLS and TEM, the size of the antigen–antibody complexes in the NanoPITA reaction can grow up to approximately 100 nm. Based on FDTD simulations, a part of the electric field will be transferred from the chip to the immune complexes, resulting in a decrease in the electric-field strength at the edge of the nanocup and a decreased transmitted light intensity and increased OD value. Furthermore, NanoPITA, the one-step assay without complicated operations, is extremely sensitive (LOD below 1 ng/mL) to the CRP within 10 min. In summary, this new method with the SPR nanochip provides great potential for detecting the formation of nanoparticles in real time, and for the ultrasensitive detection of specific protein biomarkers. However, the NanoPITA still has non-specific adsorption for real samples such as blood, which we think will be resolved when a better blocking reagent is invented. In the future, we believe that NanoPITA will be attractive for biological detection, and become a new technology platform for both early and rapid clinical diagnosis of cardiovascular diseases and other related diseases.

## Figures and Tables

**Table 1:** Limit of detection, process time and biological modification time of the CRP in various experimental methods.

Experimental Method	Experimental Readout	LOD (ng/mL)	Linear Range (ng/mL)	Process Time	Biological Modification Time
Optical fiber Bragg gratings [45]	Bragg wavelength	10	10–10^6^	10 min	11 h
Surface molecular imprinting [46]	Differential pulse voltammetry	40	180–8510	N/A	9 h
Lossy mode resonances [47]	Wavelength shift	62.5	62.5–1000	61 s	26 min
Microparticle tracking velocimetry [48]	Brownian velocity	100	100–10^4^	10 min	3 h
Surface acoustic wave [49]	Amplitude	100	100–10^6^	10 min	48.5 h
NanoPITA (this study)	Optical density	0.54	1–500	35 min	10 min

## Data Availability

Not applicable.

## Supplementary Materials

The following supporting information [51] can be downloaded at: https://www.mdpi.com/article/10.3390/bios12110958/s1.
