# Supplementary material for: Ultrasensitive Detection of C-Reactive Protein by a Novel Nanoplasmonic Immunoturbidimetry Assay
Source: Biosensors (Basel). 2022 Nov 2;12(11):958. doi: 10.3390/bios12110958 (PMC9688280; doi:10.3390/bios12110958)
Supplement: Supplementary file 1 [file biosensors-12-00958-s001.zip › biosensors-1978635-supplementary.pdf]

biosensors

MDPI

Supporting Information

Ultrasensitive Detection of C-Reactive Protein by A Novel Nanoplasmonic Immunoturbidimetry Assay

Tang Dang [1,2,†], Zhenyu Li [3,†], Liyuan Zhao [4,†], Wei Zhang [1], Liping Huang [1], Fanling Meng [4,*], Gang Logan Liu [1,*] and Wenjun Hu [1,*]

[1] School of Life Science and Technology, Huazhong University of Science and Technology, Wuhan 430022, China
[2] Department of Bioengineering, The University of Tokyo, 1-3-7 Hongo, Bunkyo-ku, Tokyo 113-8656, Japan
[3] Cancer Center, Union Hospital, Tongji Medical College, Huazhong University of Science and Technology, 1277 JieFang Avenue, Wuhan 430022, China
[4] National Engineering Research Center for Nanomedicine, College of Life Science and Technology, Huazhong University of Science and Technology, Wuhan 430074, China
* Correspondence: fanlingmeng@hust.edu.cn (F.M.); loganliu@hust.edu.cn (G.L.L.); hu_wenjun@hust.edu.cn (W.H.)
† These authors contribute equally to this work.

The relationship between NanoPITA method and immunoturbidimetry

The ultrasensitive nanoplasmonic immunoturbidimetry assay (NanoPITA) method combines the LSPR and immunoturbidimetry. Molecular agglomeration process is very similar to immunoturbidimetry, in particular, the NanoPITA method mainly includes three phase reactions occurring in the nanoplasmonic device: antigen–antibody monomer formation, antigen–antibody immunocomplex formation, and precipitate formation[1] as shown in Figure S1a. The amount of precipitate is dependent on the ratio of antibody to antigen concentration, and as illustrated in Figure S1b, there are three reaction zones: antigen excess, equivalence, and antibody excess zones[2]. The NanoPITA method is performed in the antibody excess zone, where the antibody–antigen precipitation has positive correlation with the antigen. Figure S1c shows the relationship between the transmission intensity and the concentration of antigens in the antibody excess zone.

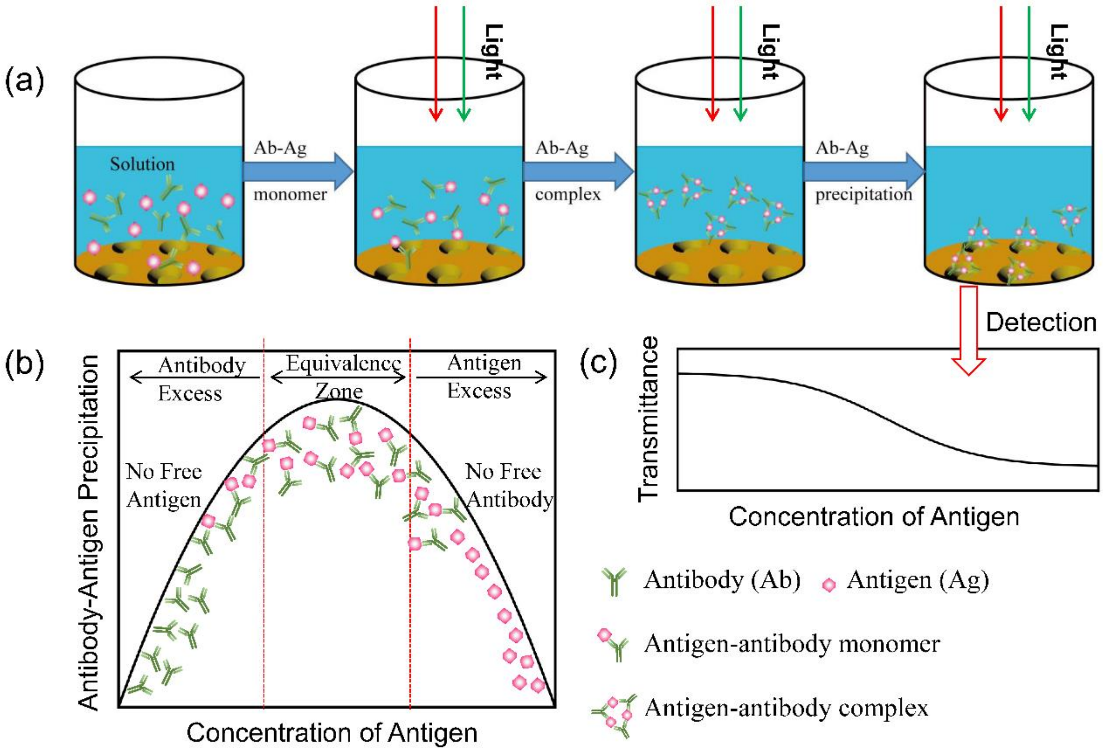

Figure S1. (a) Three phase reactions of NanoPITA method including antigen-antibody monomer formation, antigen-antibody complex formation and precipitate formation in the vicinity and inside the plasmonic nanocup array. (b) Amount of antigen-antibody precipitation as a function of antigen concentration showing antibody excess, equivalence, and antigen excess zones. (c) Relationship between light transmission out of the nanoplasmonic sensor and concentration of antigen in antibody excess zone.

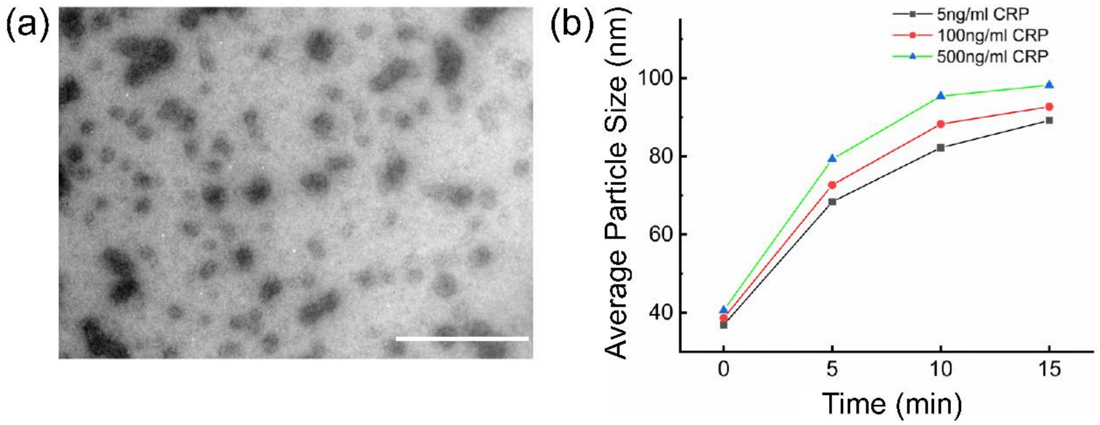

Figure S2. (a) TEM image of the immune complex after 15 minutes of 500 ng/ml CRP, anti-CRP antibody and PEG-6000 (Scale bar: 1 μm). (b) Different average particle size of different concentration of CRP and different reaction time. The size of complex was determined by dynamic light scattering analysis (Zetasizer Nano ZSP, Malvern), 3X3 measurement runs were performed at 23 °C. .

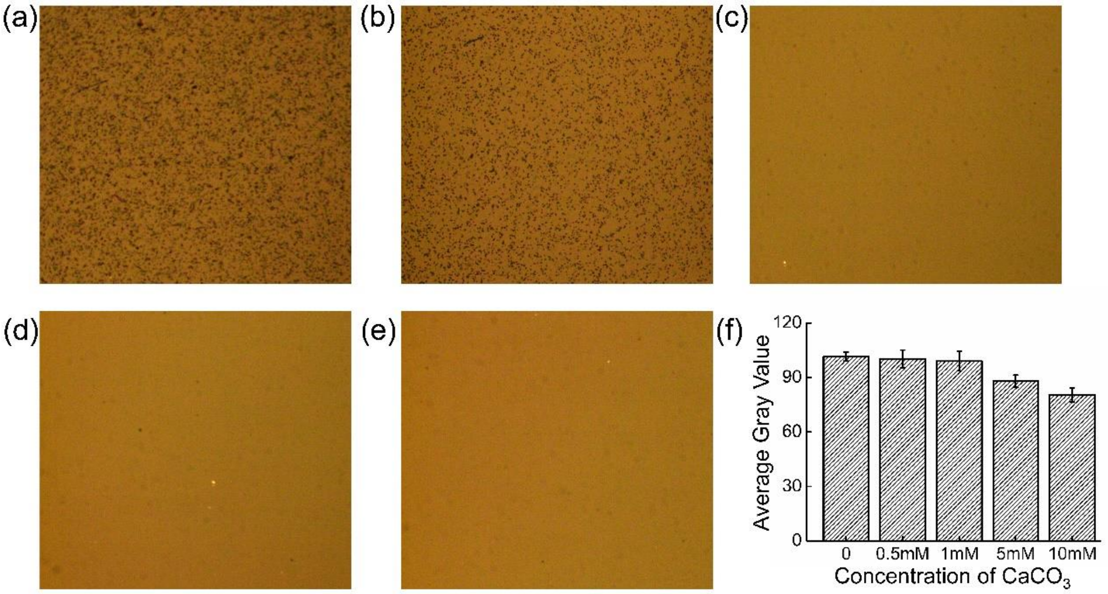

Figure S3. (**a**)–(**e**) Microscope images of 10mM Na_2CO_3 reacting with 10mM $CaCl_2$ (a), 5mM $CaCl_2$ (b), 1mM $CaCl_2$ (c), 0.5mM $CaCl_2$ (d), DDW (e) for 30 minutes, all images were taken by Olympus IX73 microscope. (**f**) Grayscale statistics of microscope images (by software imageJ). (When 10 nM Na_2CO_3 was added, the solution became cloudy after a few minutes.).

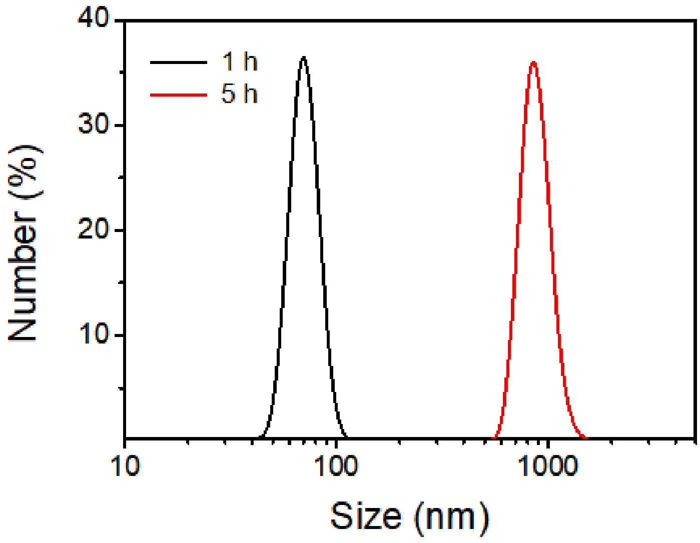

Figure S4. The particle size of 32 mM IPPA after 1 hour and 5 hours of self-aggregation reaction

Table S1. Fitting function, parameter and the coefficient of determination (R^2) for fitting for different concentration of CRP by surface plasmon resonance enhanced immunoturbidimetry method at 600nm wavelength.

[CRP] (ng/ml)	Fitting Function	Parameter A	Parameter B	R^2
0	$y = 0.00100 \times (\frac{1}{1+e^{-0.0087x}} - \frac{1}{2})$	0.00100	0.00870	0.7797
1	$y = 0.00259 \times (\frac{1}{1+e^{-0.0096x}} - \frac{1}{2})$	0.00259	0.00963	0.8779
5	$y = 0.00602 \times (\frac{1}{1+e^{-0.01339x}} - \frac{1}{2})$	0.00602	0.01339	0.8294
10	$y = 0.02162 \times (\frac{1}{1+e^{-0.01958x}} - \frac{1}{2})$	0.02162	0.01958	0.7245
100	$y = 0.02348 \times (\frac{1}{1+e^{-0.03217x}} - \frac{1}{2})$	0.02348	0.03217	0.8746
500	$y = 0.02225 \times (\frac{1}{1+e^{-0.04078x}} - \frac{1}{2})$	0.02225	0.04078	0.8904

Table S2. Fitting function, parameter and the coefficient of determination (R^2) for fitting for different concentration of CRP by traditional immunoturbidimetry method at 600nm wavelength.

[CRP] (ng/ml)	Fitting Function	Parameter A	Parameter B	R^2
0	$y = 0.00647 \times (\frac{1}{1+e^{-0.00353x}} - \frac{1}{2})$	0.00647	0.00353	0.9515
1	$y = 0.00659 \times (\frac{1}{1+e^{-0.00353x}} - \frac{1}{2})$	0.00659	0.00353	0.9719
10	$y = 0.00569 \times (\frac{1}{1+e^{-0.00426x}} - \frac{1}{2})$	0.00569	0.00426	0.9508
100	$y = 0.00662 \times (\frac{1}{1+e^{-0.00434x}} - \frac{1}{2})$	0.00662	0.00434	0.9630
500	$y = 0.00535 \times (\frac{1}{1+e^{-0.00506x}} - \frac{1}{2})$	0.00535	0.00506	0.9568
1000	$y = 0.01042 \times (\frac{1}{1+e^{-0.00681x}} - \frac{1}{2})$	0.01042	0.00681	0.9712
5000	$y = 0.01229 \times (\frac{1}{1+e^{-0.01117x}} - \frac{1}{2})$	0.01229	0.01117	0.8857
10000	$y = 0.01901 \times (\frac{1}{1+e^{-0.01649x}} - \frac{1}{2})$	0.01901	0.01649	0.9279

Table S3. Fitting function, parameter and the coefficient of determination (R^2) for fitting for different concentration of CRP by traditional immunoturbidimetry method at 340nm wavelength.

[CRP] (ng/ml)	Fitting Function	Parameter A	Parameter B	R^2
0	$y = 0.00823 \times (\frac{1}{1+e^{-0.01228x}} - \frac{1}{2})$	0.00823	0.01228	0.9862
1	$y = 0.00868 \times (\frac{1}{1+e^{-0.01142x}} - \frac{1}{2})$	0.00868	0.01142	0.9866
10	$y = 0.00813 \times (\frac{1}{1+e^{-0.01271x}} - \frac{1}{2})$	0.00813	0.01271	0.9554
100	$y = 0.00811 \times (\frac{1}{1+e^{-0.01265x}} - \frac{1}{2})$	0.00811	0.01265	0.9802
500	$y = 0.00709 \times (\frac{1}{1+e^{-0.01277x}} - \frac{1}{2})$	0.00709	0.01277	0.9656
1000	$y = 0.01269 \times (\frac{1}{1+e^{-0.02147x}} - \frac{1}{2})$	0.01269	0.02147	0.9125
5000	$y = 0.02775 \times (\frac{1}{1+e^{-0.03161x}} - \frac{1}{2})$	0.02775	0.03161	0.9301
10000	$y = 0.02981 \times (\frac{1}{1+e^{-0.04410x}} - \frac{1}{2})$	0.02981	0.04410	0.9830

Table S4. Fitting function, parameter and the coefficient of determination (R^2) for fitting for different samples by surface plasmon resonance enhanced immunoturbidimetry method at 600nm wavelength.

Sample	Fitting Function	Parameter A	Parameter B	R^2
Tris	$y = 0.00100 \times (\frac{1}{1+e^{-0.0087x}} - \frac{1}{2})$	0.00100	0.00870	0.7797
100ng/ml CRP	$y = 0.02348 \times (\frac{1}{1+e^{-0.03217x}} - \frac{1}{2})$	0.02348	0.03217	0.8746
2000ng/ml BSA	$y = 0.00119 \times (\frac{1}{1+e^{-0.00747x}} - \frac{1}{2})$	0.00119	0.00747	0.9632
2000ng/ml CEA	$y = 0.00199 \times (\frac{1}{1+e^{-0.00652x}} - \frac{1}{2})$	0.00199	0.00652	0.9183
2000ng/ml IgG	$y = 0.00279 \times (\frac{1}{1+e^{-0.00751x}} - \frac{1}{2})$	0.00279	0.00751	0.9529

References

[1] D.J. MARMER, P.E. HURTUBISE, NEPHELOMETRIC AND TURBIDIMETRIC IMMUNOASSAY, Immunoassay, Elsevier1996, pp. 363-387.

[2] J. Whicher, C. Price, K. Spencer, A.M. Ward, Immunonephelometric and immunoturbidimetric assays for proteins, CRC Critical Reviews in Clinical Laboratory Sciences 18(3) (1982) 213-260.
